# Early Postnatal Exposure to Cigarette Smoke Leads to Later Airway Inflammation in Asthmatic Mice

**DOI:** 10.1371/journal.pone.0171021

**Published:** 2017-01-30

**Authors:** Fei Huang, Hang Cheng, Yu-tong Zhang, Yang-hua Ju, Ya-nan Li

**Affiliations:** 1 Department of Orthopedics, China-Japan Union Hospital of Jilin University, Changchun, Jilin, PR China; 2 Department of Pediatrics, The First Hospital of Jilin University, Changchun, Jilin, PR China; 3 Department of Molecular Biology, Basic Medical College of Jilin University, Changchun, PR China; McGill University, CANADA

## Abstract

**Background and objective:**

Asthma is one of the most common airway inflammatory diseases. In most cases, asthma development is related to ubiquitous harmful environmental exposure factors in early-life. Previous studies have indicated that smoking can promote asthma development and increase the difficulty of asthma control. The aim of this study was to determine the effects of early-life CS exposure on ovalbumin (OVA)-sensitized asthmatic mice.

**Methods:**

Pathological and immunological functions were analyzed in an adult asthma mice model in which mice were sensitized with OVA combined with early-life CS exposure.

**Results:**

Mice exposed to CS for only 5 weeks demonstrated significantly reduced pulmonary compliance, increased airway inflammation, and augmented cellular and humoral immune responses. In addition, CS inhalation was sufficient to facilitate OVA sensitization and challenge asthmatic development. Meanwhile, CS exposure amplified regulatory T cell-mediated immunity inhibition, but still did not offset the increased effector T cell-mediated inflammatory response.

**Conclusion:**

Early-life CS exposure is significantly associated with later pulmonary injury and aggravation of T-cell immunologic derangement in asthmatic mice.

## Introduction

Asthma is one of the most common airway inflammatory diseases. The incidence of asthma has significantly increased in recent decades around the world and especially in the developed countries, which has brought considerable mental stress to patients and economic burden to societies and patients’ families [1].

In most cases, asthma development is related to exposure to ubiquitous harmful environmental factors, such as pathogens, allergenic pollen, tobacco use, air pollution, allergenic food, and special occupational exposures in early-life. Among these precipitating factors, cigarette smoke (CS) exposure has become a research hotspot for the prevention and treatment of asthma because of its universality and controllability [2, 3]. Previous studies have indicated that smoking can promote asthma severity, lessen respiratory function, and increase the difficulty of asthma control [4–6]. Smoking is a risk factor for both pediatric and adult asthma [7, 8]. The asthma aroused by a short period of CS exposure in adults has been studied for decades, and even though the pathogenesis of asthma is not entirely clarified, an excessive immune response from effector T cells including T helper 1 (Th1), Th2, and Th17 cells, are key pathogenic factors [9, 10]. We also know that a host immune disorder leads to regulatory T cell (Treg) reduction and impairment. Orton *et al* reported that most passive cigarette smokers are children of parents who smoke [11]. Nevertheless, the precise effect and the detailed mechanism of asthma promoted by early-life CS exposure have not been explored. In this study, we initially established a mouse model in which postnatal day 8 (P8) mice were exposed to CS and sensitized with ovalbumin (OVA) to induce asthma. We then explored the alterations in physiology, pathology, and immunology in these mice during adulthood. Our data suggest that CS exposure during infancy will result in long-standing deterioration of the pulmonary immune microenviroment and facilitates allergic airway inflammation in adults.

## Methods

### Animals

Newborn virgin BALB/c mice of both sexes were employed in our study. The mice were obtained from the Experimental Animal Center of Jilin University and housed in a specific pathogen-free facility with free access to food and water. All animal protocols were authorized by the Jilin University Animal Ethics Committee.

### Asthma model and grouping

**Fig 1:**
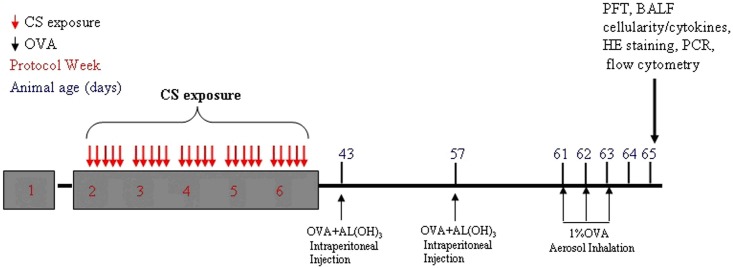
Schematic of the mouse model of early-life expose to CS and OVA sensitization. The processes of CS exposure and OVA sensitization in BALB/c mice are illustrated. The number in grey box indicates the number of weeks post-delivery. The numbers above the line indicate the time in postnatal days. The arrow represents the time of each treatment. The treatment method and time are described above or below the arrow.

To investigate whether early-life exposure to CS influences subsequent allergen OVA-induced asthma, P8 pups were exposed to CS for 5 weeks (see *CS exposure*). Fresh air was used for the negative control. To sensitize the mice to asthma, they were given an intraperitoneal injection of 10 μg OVA (grade V; Sigma-Aldrich, St. Louis, MO, USA) and 200 μg aluminum hydroxide (AL(OH)_3_) dissolved in 100 μL phosphate-buffered saline (PBS) at P43 and P57, and subjected to 1% aerosolized OVA inhalation for 20 min on P61, P62, and P63 (Fig 1). PBS injection was used as a negative control. Mice were randomly divided into four groups (n = 25 each): air exposure with PBS sensitization (air-PBS), CS exposure with PBS sensitization (CS-PBS), air exposure with OVA sensitization (air-OVA), and CS exposure with OVA sensitization (CS-OVA). Pulmonary function tests (PFTs) and BALF analysis were performed on P65. After that, the mice were sacrificed and lungs were collected for other experiments. In each group, 6 mice were used for pulmonary resistance analysis and lavage, another 6 mice were used for histological analysis, 7 mice were used for isolation of cells, and the last 6 mice were employed for flow cytometric analysis.

### Methods of euthanasia

All mice in this study were euthanatized by carbon dioxide inhalation and cervical dislocation. Briefly, mice in euthanasia chamber was euthanized by 20–30% chamber volume carbon dioxide gas flow per minute for at least one minute to induce respiratory arrest. Following, the mice cervical was dislocated to assure euthanasia.

### CS exposure

The CS exposure was performed as described previously [12] with corresponding improvements. Briefly, the mice were exposed to CS (side-stream smoke, 600 mg/m^3^ total suspended particulates, 360 ppm carbon monoxide) from a 3R4F reference cigarette passively in a whole-body exposure chamber using the PAB-S200 Animal Passive Smoking Exposure System (BioLab Technology Co. Ltd., Beijing, China). An optimal smoke/air ratio of 1:6 was obtained. Mice were exposed to CS twice daily for 1 hour each time, 5 days per week for 5 weeks.

### Pulmonary resistance analysis

Asthma is characterized by significantly increased pulmonary resistance [13]. Therefore, the status of pulmonary resistance in the CS- and OVA-induced mice asthma model was detected by dynamic lung resistance with the flexiVent forced oscillation technique (SCIREQ, Montreal, Canada). The mice were anesthetized and ventilated, and pulmonary resistance analysis was measured in response to methacholine (MCh) in increasing concentrations.

### Bronchoalveolar lavage

The BALF of the mice at P65 was collected as described previously [9, 14]. The number of viable cells in each individual BALF sample was counted upon staining with Trypan blue. Aliquots of BALF were smeared on glass slides for Wright staining. The numbers of macrophage, eosinophils, lymphocytes, and neutrophils were counted among 200 total cells per BALF sample in a blinded manner.

### Histological analysis and scoring

Lungs harvested from the mice on P65 were fixed in 4% formalin for a minimum of 24 hours and paraffin-embedded. Lung sections (2–3 μm) were stained with hematoxylin–eosin (HE) for histological assessment via light microscopy by two independent blinded investigators. Three random fields were counted per section, with 2 sections per mouse and 6 mice per group. Peribronchial and airway cells were graded [15] as: 0, no cells; 1, a few cells; 2, a ring of cells 1 cell layer deep; 3, a ring of cells 2–4 cell layers deep; and 4 with a ring of cells more than 4 cell layers deep.

### Flow cytometric analysis

Single-lung cell suspensions were prepared and enriched by nylon wool from mice lungs. Following a previously described protocol [16, 17], the frequency of Treg (CD4^+^ Foxp3^+^) cells and effector T cells T_h_1 (CD3^+^CD8^−^interferon [IFN]-γ^+^), T_h_2 (CD3^+^CD8^−^IL-4^+^), T_h_9 (CD3^+^CD8^−^IL-9^+^), and T_h_17 (CD3^+^CD8^−^IL-17A^+^) were calculated in individual samples. Cells were stained with the following antibodies (all from eBioscience): phycoerythrin (PE)-conjugated anti-FOXP3, fluorescein isothiocyanate (FITC)-conjugated CD4; PE-conjugated CD3; allophycocyanin-conjugated CD8, FITC-conjugated interferon (IFN)-γ, interleukin (IL)-4, IL-9, and IL-17A. Homotype-independent antibody was employed as the negative control. IFN-γ, IL-4, IL-9, and IL-17A intracellular staining was carried out after 4-h treatment with 25 ng/mL phorbol 2-myristate 13-acetate, 1 mg/mL ionomycin, and 2 nmol/mL monensin (all from Sigma-Aldrich).

### Real-time PCR for Foxp3 analysis

Total RNA was isolated from lung tissue using TRIzol reagent (Invitrogen, Carlsbad, CA, USA). The mRNA expression of the *Foxp3* and the internal reference mouse glyceraldehyde phosphate dehydrogenase (*Gapdh*) genes was detected by SYBR Green-based quantitative real-time PCR with MMLV reverse transcriptase (Promega, Madison, WI, USA). The primers for RT-PCR analysis were as follows: *Foxp3*: (sense, S) 5′-CTTATCCGATGGGCCATCCTGGAAG-3′ and (antisense, A) 5′-TTCCAGGTGGCGGGGTGGTTTCTG-3′ (112-bp product); *Gapdh*: (S) 5′-GCACAGTCAAGGCCGAGAA-3′ and (A) 5′-CCTCACCCCAT TTGATGTTA GTG-3′ (96-bp product). The RT reaction was processed at 70°C for 5 min, 42°C for 60 min, and 95°C for 5 min. The PCR reaction was carried out at 95°C for 30 s, 58°C for 30 s, and 72°C for 30 s for each cycle with 40 cycles run in total. The results were analyzed using the comparative threshold cycle value (2^-ΔΔCT^) method [18].

### Western blotting for Foxp3 protein expression

The proteins extracted from lung tissues were separated on 10% sodium dodecyl sulfate (SDS)-polyacrylamide gel electrophoresis (PAGE) gels and transferred onto nitrocellulose membranes. Subsequently, the membranes were incubated with goat anti-mouse Foxp3 (1:50 dilution) or β-actin antibodies (1:500 dilution, Santa Cruz Biotechnology, Santa Cruz, CA, USA). The probed membrane was then reacted with horseradish peroxidase-conjugated rabbit anti-goat or anti-mouse IgG (1:1000 dilution, Santa Cruz Biotechnology). The probed proteins (Foxp3 or β-actin) were finally visualized by enhanced chemiluminescence (Santa Cruz Biotechnology). Protein expression was analyzed quantitatively by optical densitometry.

### Cellular proliferation assay

The Treg suppressive activity was evaluated by the [^3^H]-thymidine incorporation method. The fresh CD4^+^CD25^+^ Treg subset and the CD4^+^CD25^−^ responder T-cell subset were isolated from the lungs of untreated mice, and the induced CD4^+^CD25^+^ Tregs were isolated from the lungs of CS-PBS mice. All the CD4^+^CD25^+^ and CD4^+^CD25^−^ T cells were purified with a MACS Treg Isolation Kit (Miltenyi Biotec, Auburn, CA, USA). The T-cell mixtures were adjusted to (2 × 10^5^) different ratios of (CD4^+^CD25^−^) T cells and (CD4^+^CD25^+^) Tregs at 1:1, 2:1, 4:1, 8:1, or only T cells (positive control) in 96-well plates. These cells were incubated at 37°C in 5% CO_2_ for 72 h, and exposed to the [^3^H]-thymidine pulse (1 μCi/mL) for another 8 h. Finally, the [^3^H]-thymidine-incorporating (proliferated) cells were detected by scintillation.

### Enzyme-linked immunosorbent assay (ELISA) for cytokine and OVA-specific immunoglobulin expression

The activities of IL-4, IL-5, IL-13, IL-9, IL-21, IL-23, IL-17A, and IFN-γ in the supernatants of the co-culture medium were determined corresponding commercially available double-antibody sandwich ELISA kits and (all from eBioscience). Serum was isolated from whole blood, and the total IgE and IgG1 concentrations were measured by ELISA kits. OVA-Specific IgE and IgG1 were detected with the Mouse OVA-specific IgE ELISA Kit and Mouse OVA-specific IgG1 ELISA Kit (both from Biolegend). All operations were performed in accordance with the manufacturer’s instructions.

### Statistical analysis

All data are shown as the mean ± standard deviation (SD). The differences between control and testing groups were calculated by analysis of variance using SPSS 18.0 for Windows. *p* < 0.05 was considered statistically significant.

## Results

### Establishment of asthmatic mouse model and sample collection

To establish a CS-exposed mouse model of asthma, P8 pups were exposed to CS for 5 weeks and sensitized with OVA from P43. The asthma model was established at P63 (Fig 1). All the samples for the next analysis were collected on P65.

### CS exposure aggravated airway inflammation

**Fig 2:**
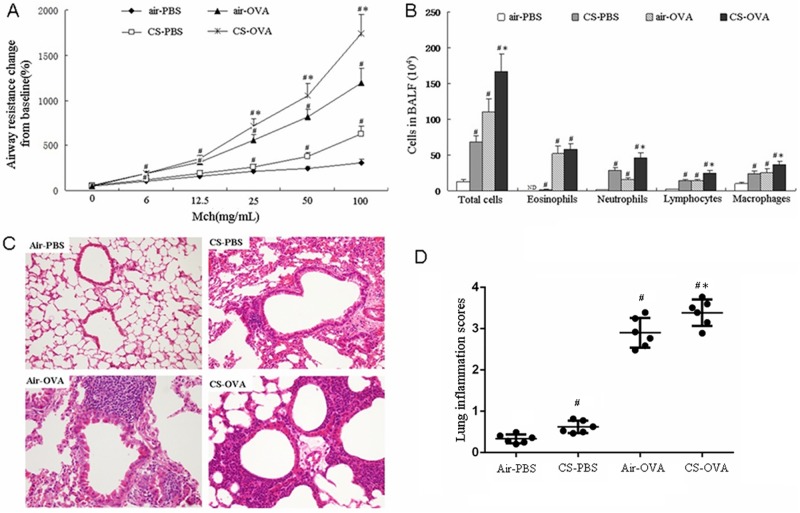
Early-life CS exposure aggravated asthmatic airway inflammation. (A) Pulmonary resistance was analyzed with the flexiVent forced oscillation technique (SCIREQ). Pulmonary resistance was measured in response to methacholine (MCh) at the indicated concentration in the air exposure with PBS sensitization (air-PBS), CS exposure with PBS sensitization (CS-PBS), air exposure with OVA sensitization (air-OVA), and CS exposure with OVA sensitization (CS-OVA) groups. The percentage rates of the baseline were normalized to 100. (B) The numbers of eosinophils, neutrophils, lymphocytes, macrophages, and total cells were counted in the BALF of the air-PBS, CS-PBS, air-OVA, and CS-OVA groups. (C) HE staining of lung sections from the air-PBS (upper left), CS-PBS (upper right), air-OVA (lower left), and CS-OVA (lower right)-treated mice. (D) The degree of inflammation of the groups in (C) was analyzed by scoring the cells around the airways (0, no cells; 1, a few cells; 2, a ring of cells 1 cell layer deep; 3, a ring of cells 2–4 cell layers deep; and 4 with a ring of cells more than 4 cell layers deep) in the four groups. All values are the means ±SD of the results from 6 mice per group. ^#^*P*<0.05 vs. Air-PBS group; *P<0.05 vs. Air-OVA group.

In the model mice, pulmonary function was analyzed by pulmonary resistance measurement (Fig 2A). The resistance volume in the air-OVA and CS-OVA groups was augmented along with an increase in the concentration of MCh, which was significantly higher than that in the air-PBS group. In contrast, the resistance in the CS-PBS group was similar to that in the air-PBS group with a low concentration of MCh (0–2.5 mg/mL) but obviously increased with a higher concentration of MCh (100 mg/mL). The resistance in the CS-OVA group was significantly greater than that in the air-OVA group. Therefore, early-life CS exposure increased pulmonary resistance, which suggested it aggravated allergic airway resistance in normal and asthmatic mice (CS-PBS and CS-OVA).

Subsequently, the BALF was analyzed via cytology (Fig 2B). Compared with that in the air-PBS group, the total cell count as well as the counts of eosinophils, neutrophils, lymphocytes, and macrophages were increased in the CS-exposed and OVA-sensitized groups. CS exposure primarily increased the neutrophil count, whereas OVA sensitization generally increased the number of eosinophils. Thus, early-life CS exposure could promote OVA-induced cellular aggravation.

In addition, the histochemical properties of mouse airways after CS and OVA treatment were determined by HE staining (Fig 2C) and lung inflammatory scores (Fig 2D). Characteristics of inflammatory infiltration, including multiple pulmonary goblet cells, airway epithelial hyperemia, and edema, were observed in OVA-treated mice (air-OVA). The CS-treated group (CS-PBS) showed relatively moderate inflammatory infiltration. CS exposure (CS-OVA) aggravated OVA-induced inflammatory infiltration reactions (higher degree of airway epithelial hyperemia and edema, more pulmonary goblet cells).

Finally, the general degree of inflammation in the CS- and OVA-treated mice was scored according to a previously described method [15].

### Early-life CS exposure increased Treg activity in asthmatic mice

**Fig 3:**
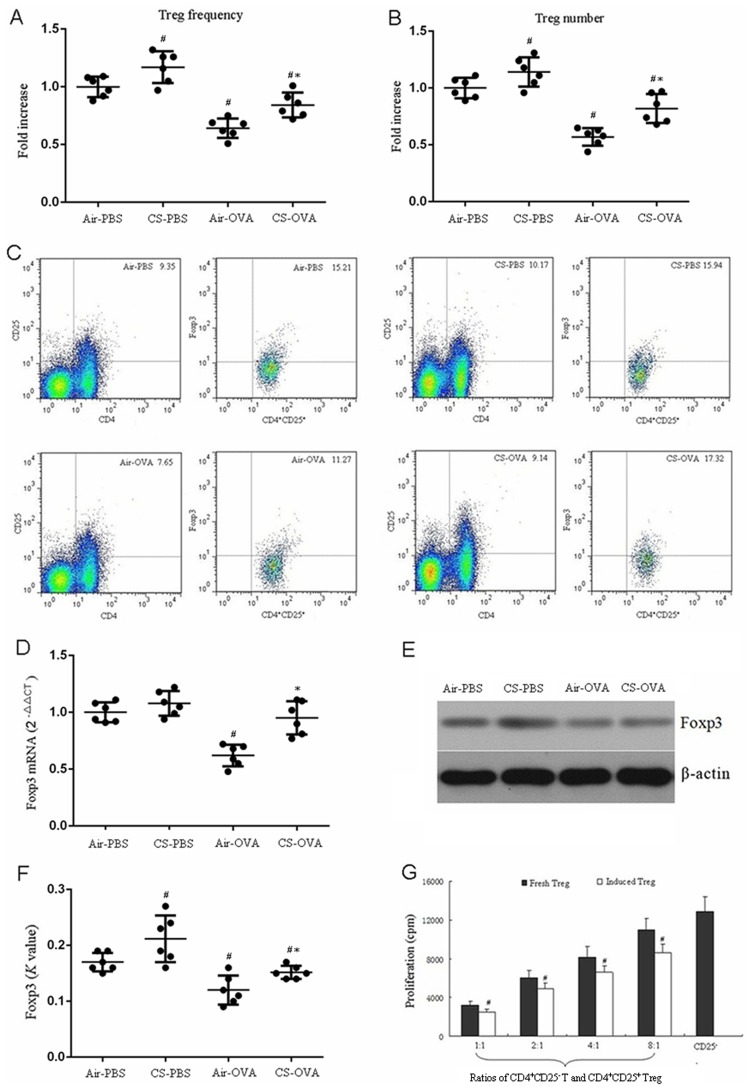
Early-life CS exposure induced Treg-mediated immune suppressive activity. (A-C) The population of CD4^+^CD25^+^ Tregs of lung tissue was analyzed by flow cytometry. The Treg frequency (A) and number (B) were determined from the data shown in (C). Foxp3 expression of lung tissue was estimated by RT-PCR (D) and western blotting (E-F). The proliferation of lung T cells at different ratios of CD4^+^CD25^-^ to CD4^+^CD25^+^ were assayed by the [^3^H]-thymidine incorporation method. The fresh Tregs and the induced Tregs were isolated from the lung of untreated mice and CS-PBS mice, respectively. (G) The values are the means ±SD of the results from 6 mice per group. ^#^*P*<0.05 vs. Air-PBS group; *P<0.05 vs. Air-OVA group in (A, B, D and F). ^#^*P*<0.05 vs. Fresh Tregs in (G).

The amount and activity of Tregs were investigated in CS- and OVA-treated mice (Fig 3). The number and frequency of Tregs in CS-PBS mouse lungs were markedly greater than in air-PBS mice. Nonetheless, it was significantly decreased in air-OVA mice. The Treg number was markedly increased in CS-OVA mice compared with air-OVA mice and less than that in the air-PBS group (Fig 3A–3C). Foxp3 is an important regulating factor of Tregs [19]. Therefore, Foxp3 expression at the transcription and protein levels in lungs were detected by RT-PCR and western blotting, respectively (Fig 3D–3F). The alteration pattern of the mRNA and protein levels of Foxp3 in the observed groups were consistent with that of the Treg numbers and frequency (Fig 3A and 3B). Therefore, induction of Tregs by early-life CS exposure was examined in comparison to that in air-PBS mice. In addition, the immune suppressive activity was augmented along with the increased proportion of CD4^+^CD25^+^ Tregs, and the maximal inhibitory activity was detected at a 1:1 ratio of CD4^+^CD25^+^ to CD4^+^CD25^-^ cells (Fig 3G).

### Early-life CS exposure exacerbated immunity

**Fig 4:**
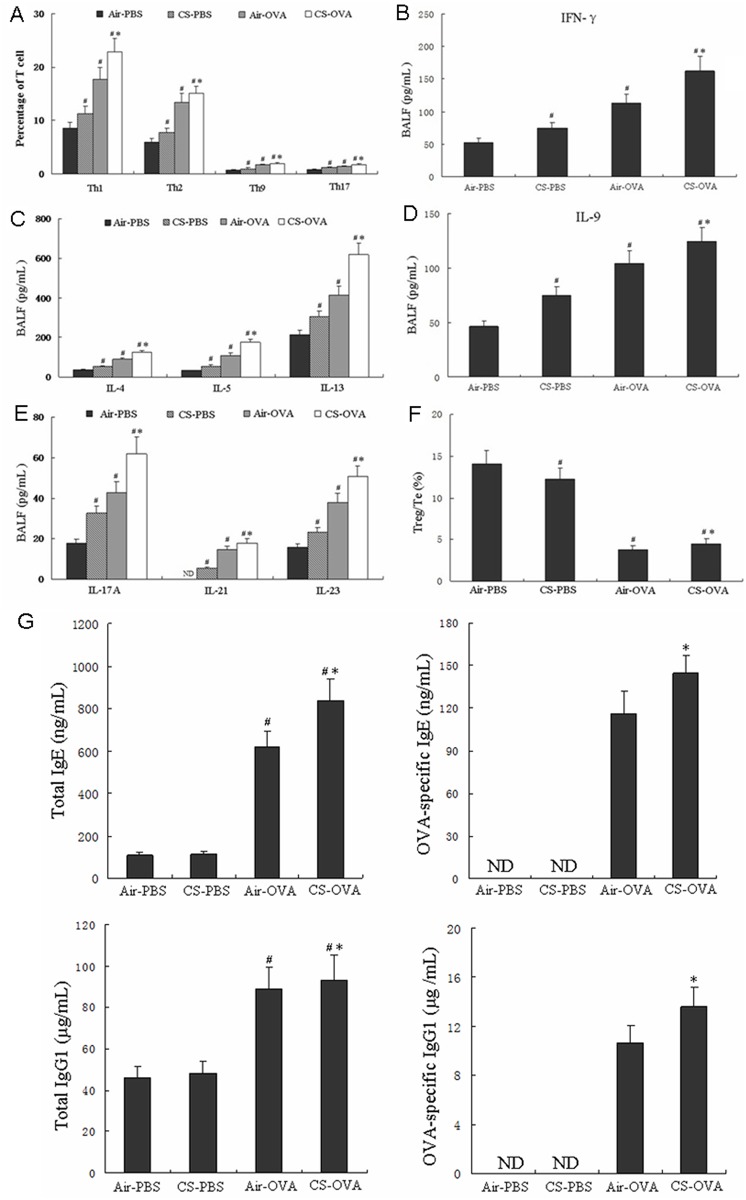
Early-life CS exposure suppressed cellular immunity. (A) The percentage of (CD4^+^CD25^+^) Tregs was analyzed in lungs of the air-PBS, CS-PBS, air-OVA, and CS-OVA-treated mice. The cytokines INF-γ (B), IL-4, -5, and -14 (C), IL-9 (D), and IL-17, -21, -23 (E) were detected in BALF samples from the air-PBS, CS-PBS, air-OVA, and CS-OVA groups. The ratio of Treg/effector T cells (%) in the lungs were calculated in F. (G) Serum antibody concentrations were determined by ELISA in the same groups as above. All data were obtained by flow cytometric analysis, and the values are the means ±SD of the results from 6 mice per group. ^#^*P*<0.05 vs. Air-PBS group; *P<0. 05 vs. Air-OVA group.

Abnormal lymphocyte activity has been reported previously in both allergic asthma and with CS exposure [9, 20, 21]. Thus, we observed lymphocyte activities in the lungs by flow cytometry and cytokine expression in BALF in the early-life CS exposure and OVA-induced asthma model (Fig 4). The percentages of Th1, Th2, Th9, and Th17 cells were greater in the CS-PBS and Air-OVA mice than in the Air-PBS mice. Hence, early-life CS exposure could statistically increase these Th cell populations in the CS-OVA group (*p*<0.05 compared to the Air-OVA mice, Fig 4A). As expected, the levels of cytokines produced by Th1 (IFN-γ), Th2 (IL-4, IL-5, IL-13), Th9 (IL-9), and Th17 cells (IL-17A, IL-21, IL-23) were all significantly increased in the CS-PBS and air-OVA mice compared with the control (air-PBS) mice (Fig 4B–4E). The maximal levels of the observed Th cell-related cytokines were detected in CS-OVA mice and found at levels higher than those in the air-OVA mice (Fig 4B–4E). The ratio of Tregs/effector T cells in the CS-PBS group and air-OVA group was significantly lower than control (air-PBS) mice. The ratio of Tregs/effector T cells in the CS-OVA group was considerably lower than that in the CS-PBS group as well, but higher than that in the air-OVA group (Fig 4F). The total serum IgE and IgG1 concentrations did not differ between air-PBS and CS-PBS mice, but were significantly elevated in the air-OVA and CS-OVA groups compared with CS-PBS mice, and the OVA-specific IgE and OVA-specific IgG1 concentrations in the air-OVA group were significantly increased in the serum, compared with that in CS-PBS mice. This level was further increased after combined exposure to cigarette smoke in early life (Fig 4G).

## Discussion

CS contains more than 4500 compounds and pollutants that can directly induce asthma. Inhalation of CS can hurt the airway epithelial cells, reduce ciliary movement, and increase airway resistance and glandular secretion [3, 22]. It was reported that smoking by adults can increase the asthma morbidity, and smoking by pregnant women will raise the risk of asthma of their offspring [23]. In addition, passive smoking can increase the incidence of asthma in adolescents. Therefore, reducing CS exposure is an effective measure to prevent asthma [24]. It is worth noting that, although current studies found that passive smoking by infants can significantly increase the probability of asthma in the future, the specific mechanism is not completely clear. Most previous studies were focused on the effect of smoking on the development of asthma in adulthood. However, the effect of early-life passive CS exposure on the development of asthma in future life is undefined and was explored in the present study. We employed a mouse model in a pure experimental environment to expose infant mice to CS and sensitized the mice to asthma by OVA in adults. The physiological and pathological properties and T-cell subsets responsible for immune activity were then analyzed in the asthmatic mice in adulthood. In our observation, early-life CS exposure alone augmented pulmonary resistance, which is a characteristic of asthma (Fig 2A), gave rise to respiratory inflammation, and increased neutrophil cells in BALF in adult mice compared to the negative control (air-PBS) (Fig 2B–2D). These results not only confirmed the above findings that CS can induce asthma and pulmonary inflammation directly, but also indicated that infant CS exposure will result in respiratory disorders by adult stage. Similar findings were reported previously that cigarette smoke exposing to early perinatal and/or postnatal mice could result in Th2 polarization and asthma, which could abnormally interfere lung development, mucociliary clearance, and Th1 activity[25]. Furthermore, Herbert *et al* found that the epithelial damage in early life by ambient environmental particulates could encourage the asthmatic inflammation in ovalbumin-sensitized mice[26]. Additionally, we revealed that CS exposure aggravated OVA-induced asthma (pulmonary compliance prohibition), airway inflammation, and increased infiltration of neutrophils, eosinophils, and other inflammatory cells compared to the CS-PBS group (Fig 2B–2D). These finding suggested that early CS exposure will intensify other disease-related factors of the respiratory system by the adult stage. This finding is in agreement with previous results for CS exposure during asthma development [27, 28].

Prior studies showed that passive smoking may increase the risk of asthma through the following mechanisms: CS exposure causes a Th2 advantage of the immune response, leading to Th1/Th2 immune imbalance, which results in the chronic airway inflammation of asthma, neutrophilic inflammation, and aggravation of asthma airway inflammation [28, 29]. CS exposure increases leukotriene secretion, which is a crucial factor in the pathogenesis of asthma, and causes airway smooth muscle contraction, eosinophil recruitment, and airway epithelial cell, fiber cell, and goblet cell proliferation [30, 31]. To explore the mechanism of the early-life exposure of CS and OVA-induced asthma, we examined T-cell related cytokines and factors in the CS-PBS, air-OVA, and CS-OVA groups. Our results displayed that early-life exposure to CS alone significantly increased the effect of T-cell subsets (Fig 4A–4E) on airway inflammation, which confirmed the previous findings of Lanckacker [27]. Moreover, CS exposure upregulated the pulmonary Treg percentage compared with that in the air-exposed mice (Fig 3A) and activated Tregs (Fig 3G) to suppress the immune response compared to that of fresh Tregs. Previous studies [32, 33] indicated that CS exposure increases the amount of airway Tregs, presumably as a self-protective mechanism of the body in which Treg numbers are elevated to prevent T-cell subset-related immune inflammation. Even though, the date from Barrett *et al* suggested the cigarette smoke induced airway hyper-responsiveness is not related to ovalbumin-specific Ig [34]. In our observation, the induction effect of early-life CS exposure on the effector T cells (Fig 4A–4E) was stronger than that on Tregs (Fig 3A and 3B), which gave rise to an abnormal population of Tregs and altered the balance of effector T cells and Tregs to make effector T cells the predominant type. Therefore, deep and extensive study is still necessary.

In addition to promoting airway inflammation (Fig 2), OVA sensitization inhibited Treg activity (Fig 3A and 3B), activated T-cell subsets (Th1, Th2, Th9, and Th17, Fig 4A–4E), resulting in a shift in the Treg/effector T cell ratio toward more effector T cells (Fig 4F). We further found that, compared with the air-OVA mice, those subjected to early-life CS exposure showed significantly aggravated asthmatic airway inflammation, increased numbers of neutrophils, lymphocytes, and macrophages in the BALF, T-cell subset activation, and secretion of the cytokines (Figs 2–4). CS- and OVA-treatment caused Th1 cells (Fig 4A and 4B) to trigger the cellular immune system and Th2 cells to arouse the humoral immune system to participate in the inflammation processes. Increased numbers of Th9 cells are strongly associated with asthmatic disorders, indicating that IL-9 can exacerbate the immune activity by inducing antibody production to promote immune cell infiltration and activity within the respiratory tract [35]. The Th17 differentiating signals actually prevent Treg differentiation [36]. In our experiments, OVA-stimulation prohibited Treg differentiation (Figs 3A, 3B and 4F). In the meantime, it amplified the Th17 differentiation, which was verified by increases in the Th17 cell-secreted cytokines, IL-17A, IL-21, and IL-23 (Fig 4E). These data indicate that OVA-induced T_h_17 differentiation prohibited Treg development.

In the investigation of inflammatory cells in BALF, CS exposure resulted in a significant increase in the number of neutrophils in BALF; in contrast, OVA sensitization alone created eosinophils (Fig 2B). OVA combined with CS exposure caused significant increases in the total cells, neutrophils, eosinophils, lymphocytes, and macrophages, compared with those in the control (air-PBS) mice. This indicated that a synergistic activity between CS and OVA directly impacted airway inflammation. It was reported that IL-17 secreted from Th17 cells recruits neutrophils. Infiltration of excess neutrophils into the lung will cause interstitial structural damage and airway injury, which results in a persistent inflammatory response [37]. The Th17 differentiating signals actually work to prevent Treg differentiation [36]. In addition, CS exposure in early life could aggravate the humoral immune response during later sensitization with OVA in adults, which is similar to the findings of previous studies [27,38]. Currently, several trials concerning asthma and cigarette smoke exposure are underway. Some studies using mouse models have focused on the effect of smoking on adult asthma mice. In some of the basic research studies, all of the observations were focused on adults [27]. Since the study concerning adult asthma influenced by early-life CS exposure is limited, the aim of this study was to determine the effects of early CS exposure on asthma in adults. Thus, young mice were passively exposed to CS, and the asthma effect was observed in adults. Previous studies found that early exposure to CS can increase the instant inflammatory response and airway responsiveness. However, the detailed mechanism of the effects of early CS exposure on asthma development in adult mice has not been extensively and deeply explored yet [39,40]. In this study, the mice were exposed to CS for 5 weeks, and then the OVA-sensitized model was constructed to detect the airway inflammation and airway reactivity in mice.

In conclusion, our study indicated that early-life CS exposure influences the function of the respiratory system in adults. CS exposure alone induced pulmonary inflammation, asthma symptoms (such as pulmonary compliance decline), and cellular and humoral immune system disorders. In addition, it exacerbates OVA-induced asthma and asthma-related pathological and immunological changes. These results remind us again that avoiding early-life CS exposure can reduce the asthmatic morbidity in future life. It also provides a theoretical mechanism for the prevention and treatment of asthma.
